# Proteomics Research on the Protective Effect of Mangiferin on H9C2 Cell Injury Induced by H_2_O_2_

**DOI:** 10.3390/molecules24101911

**Published:** 2019-05-17

**Authors:** Wei Guan, Yan Liu, Yuan Liu, Qi Wang, Hong-Liang Ye, Yan-Gang Cheng, Hai-Xue Kuang, Xi-Cheng Jiang, Bing-You Yang

**Affiliations:** 1 Key Laboratory of Chinese Materia Medica, Ministry of Education of Heilongjiang University of Chinese Medicine, Harbin 150040, China; myguanwei1234@yeah.net (W.G.); lifeliuyan@hljucm.net (Y.L.); flyliuyuan@163.com (Y.L.); 15645183280@163.com (H.-L.Y.); chengyg1992@163.com (Y.-G.C.); hxkuang@yahoo.com (H.-X.K.); 2 Department of Medicinal Chemistry and Natural Medicine Chemistry, College of Pharmacy, Harbin Medical University, Harbin 150036, China; mydearmumu@163.com

**Keywords:** iTRAQ, proteomics, mangiferin, H9C2, oxidative stress, myocardial ischemia and reperfusion injury

## Abstract

Cardiovascular disease is one of the leading causes of morbidity and mortality worldwide. Mangiferin is a natural glucosylxanthone with antioxidant and anti-inflammatory properties, which has been confirmed to protect cardiac cells from myocardial infarction and myocardial ischemia reperfusion injury (MIRI); however, the underlying mechanism is still unclear. As oxidative stress is a major pathogenesis of MIRI, an H9C2 cell injury induced by hydrogen peroxide (H_2_O_2_) was established to simulate MIRI in vitro. Herein, the protective effect of mangiferin against MIRI was evaluated and the isobaric tags for relative and absolute quantitation (iTRAQ)-based proteomics was applied to explore the underlying molecular mechanism. In this research, mangiferin markedly ameliorated the oxidative imbalance by increasing the antioxidative capacity of the H9C2 cell. Moreover, proteomics analysis revealed that mangiferin pretreatment brought twenty differently-expressed proteins back to normal, most of which were related to glucose and fatty acid metabolism. Glycolysis, citrate cycle, and fatty acid degradation pathways were highlighted by Kyoto Encyclopedia of Gene and Genomes (KEGG) analysis. Western blot validation of six cardiac metabolism-related proteins were consistent with the proteomics analysis. Taken together, mangiferin protected the cardiomyocytes from MIRI by enhancing the antioxidant capacity and increasing the activities of glycolysis, citrate cycle, and fatty acid degradation pathways.

## 1. Introduction

Myocardial infarction (MI) has been emerging as a common cardiovascular disease with high morbidity and mortality worldwide. In order to alleviate MI, thrombolytic agents and angioplasty approaches are applied to remove the obstruction in blood. However, the restoration of blood flow will lead to the damage of ischemia tissue and cause cell death, which is called myocardial ischemia reperfusion injury (MIRI) [1,2]. The pathogenesis of MIRI is complex, including oxidative stress, ion accumulation, mitochondrial membrane potential dissipation, endothelial dysfunction, immune response, and some other reasons; however, the molecular mechanism underlying MIRI is still not well revealed [3,4,5,6]. Oxidative stress dominates MIRI for a large extent [7]. Excessive reactive oxygen species (ROS) accumulate by oxidative stress during MIRI, such as hydrogen peroxide (H_2_O_2_), superoxide (O_2_^•−^), hydroxyl radical (^•^OH), and singlet oxygen (^•^O). Excessive generation of ROS disturbs mitochondrial membrane potential and results in mitochondrial dysfunction [8]. It is imperative to discover an effective compound to improve the resistance of cardiomyocyte against MIRI.

Mangiferin, is a natural C-glucoside xanthone with the molecular formula C_19_H_18_O_11_ that exists in herbal medicines and fruits [9,10,11]. Mangiferin possesses various pharmacological activities, including antioxidant, cardiac protection, anti-diabetic, anti-hyperuricemia, anti-hypertensive, anti-inflammatory, anti-microbial, and anti-obesity [12,13]. As an antioxidation agent, mangiferin attenuates oxidative stress-induced diabetic nephropathy and protects BRL cell lines from oxidative injury [14,15]. Previous research indicates that mangiferin increases the antioxidative capacity of cardiac tissue and protects cardiac myocytes during MIRI; however, the underlying mechanism remains unknown [16,17].

Proteomics, as an important research technique, has been widely used in pharmacological research of cardiovascular disease on a large scale, and high-resolution mass spectrometry proteomics has gained great progress in recent years [18,19]. Due to the high stability and sensitivity, isobaric tags for the relative and absolute quantitation (iTRAQ) technique has been applied in the field of proteomics to identify more potential biomarkers and develop novel therapeutic schemes of cardiovascular disease [20]. In this research, H_2_O_2_ was used to reproduce the MIRI cell model in the H9C2 cell line, and iTRAQ-based proteomics was applied, for the first time, to screen new biomarkers and elucidate the therapeutic mechanism of mangiferin against MIRI at the protein level. This research may provide a comprehensive mechanism of mangiferin on MIRI.

## 2. Results

### 2.1. Mangiferin Protected the H9C2 Cell Injury Induced by H_2_O_2_

**Figure 1:**
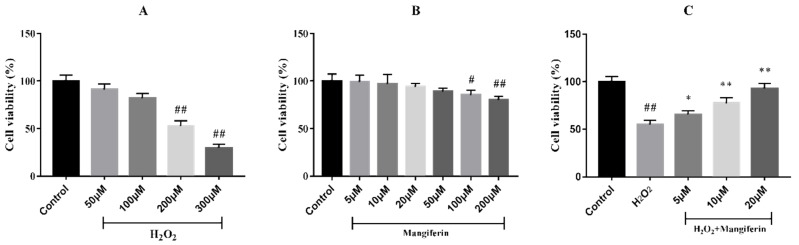
Effect of mangiferin on H9C2 cell viability after treatment with H_2_O_2_. (**A**) Effect of H_2_O_2_ on H9C2 cell viability, (**B**) Cytotoxicity of H9C2 cell viability, (**C**) Mangiferin on H_2_O_2_ induced H9C2 cell viability. ^##^ *p* < 0.01 versus the control; * *p* < 0.05 and ** *p* < 0.01 versus the H_2_O_2_ group.

The 3-(4,5-Dimethylthiazol-2-yl)-2,5-diphenyltetrazolium bromide (MTT) assay was used to evaluate the effect of mangiferin on H_2_O_2_-induced H9C2 cell injury. The results elicited that the viabilities of the cells exposed to 50 and 100 μM H_2_O_2_ showed no significant difference relative to the control group, whilst 200 and 300 μM H_2_O_2_ reduced the cell viability (Figure 1A). However, 300 μM H_2_O_2_ caused severe cell damage, thus the H_2_O_2_ concentration of 200 μM was selected for the subsequent experiments. At the same time, mangiferin caused no damage to the cells with the increasing concentrations of mangiferin up to 50 μM, and the H9C2 cell viability declined with concentrations greater than 100 μM compared to the control (Figure 1B). Then, the protective effect of mangiferin was evaluated on H9C2 cell injury induced by H_2_O_2_. The results elicited that mangiferin pretreatment obviously protected the cell injury in a dose-dependent manner (Figure 1C).

### 2.2. The Anti-Oxidation Effect of Mangiferin

**Figure 2:**
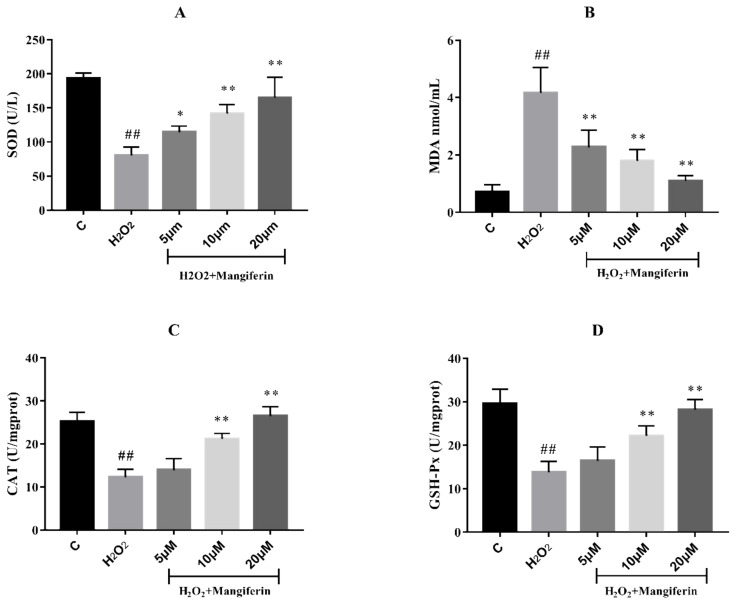
The anti-oxidative effect of mangiferin on H_2_O_2_ induced H9C2 cells. (**A**) SOD activity, (**B**) MDA content, (**C**) CAT activity, (**D**) GSH-Px activity. ^##^ *p* < 0.01 versus the control; * *p* < 0.05 and ** *p* < 0.01 versus the H_2_O_2_ group.

In order to verify whether mangiferin possesses anti-oxidation capacity, the superoxide dismutase (SOD), malonaldehyde (MDA), catalase (CAT), and glutathione peroxidase (GSH-Px) levels between the three groups were assessed by ELISA assay. The results showed that H_2_O_2_ significantly reduced the SOD, GSH-Px, and CAT levels in H9C2 cells, and simultaneously enhanced the MDA production compared with the control group. Mangiferin could decrease the MDA concentrations and increase the activities of SOD, CAT, and GSH-Px in H9C2 cells induced by H_2_O_2_ (Figure 2).

### 2.3. Effect of Mangiferin on Protein Expression Profiles in H9C2 Cell Induced by H_2_O_2_

**Figure 3:**
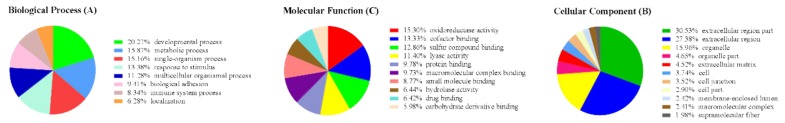
Gene ontology analysis of differently-expressed protein after mangiferin treatment. (**A**) Biological process, (**B**) cellular component, (**C**) molecular function.

**Figure 4:**
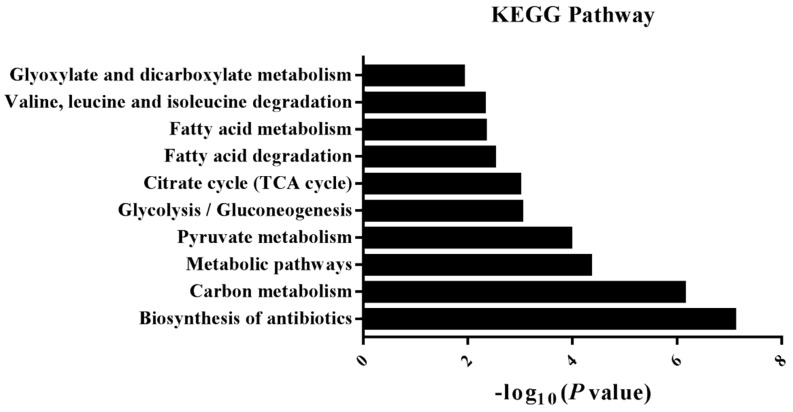
KEGG analysis of significantly enriched pathways in mangiferin treated group.

**Figure 5:**
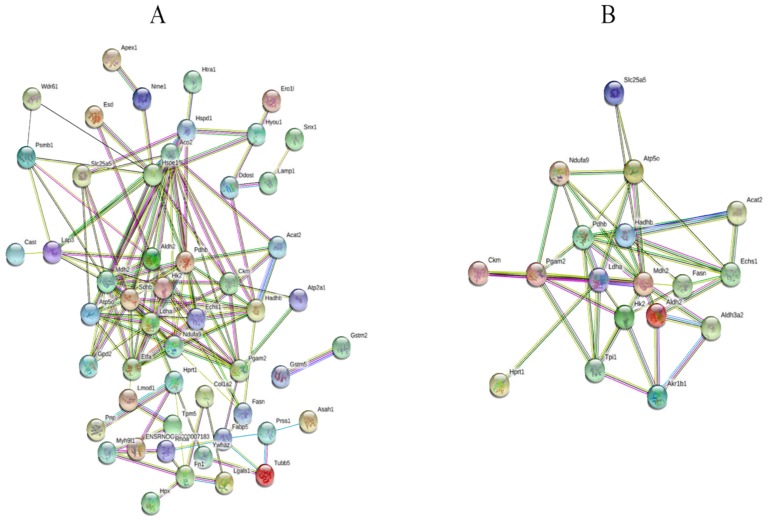
Protein interaction analysis of differently-expressed proteins by STRING. (**A**) All the differently-expressed proteins in the mangiferin treated group, (**B**) the proteins returned to normal pretreated with mangiferin.

iTRAQ-based proteomics were employed to explore the therapeutic mechanism of mangiferin against H9C2 cell injury induced by oxidative stress. A total of 1140 proteins were identified, among which 830 proteins containing at least one unique peptide with less than 1.3 unused values. The proteins with more than two peptides and fold-changes >1.25 or <0.8 were regarded as the differently-expressed proteins (DEPs) (*p* < 0.05). The results demonstrated that 69 proteins were selected as the DEPs in both H_2_O_2_ and H_2_O_2_ + mangiferin groups (Tables S1 and S2). To make a comprehensive understanding on the functional classifications of all the DEPs, DAVID bioinformatics resource 6.8 and Kyoto Encyclopedia of Gene and Genomes (KEGG) were used to categorize the DEPs based on gene ontology (GO) and KEGG pathways. The results of GO annotation revealed that a majority of DEPs were involved in the development process (20.27%), metabolic process (15.87%), single-organism process (15.16%), and response to stimulus (13.38%) in the biological process category (BP) (Figure 3A); extracellular region part (30.53%), extracellular region (27.38%), organelle (15.96%), and organelle part (4.65%) in cellular component category (CC) (Figure 3B); and oxidoreductase activity (15.30%), cofactor binding (13.33%), sulfur compound binding (12.86%), and lyase activity in molecular function category (MF) (Figure 3C). In this research, KEGG pathway analysis revealed that most of the DEPs participated in the metabolism-related pathways including glucose, fatty acid, and amino acid metabolism (Figure 4). To further explore the interaction of these identified DEPs, STRING analysis was conducted in this research, which demonstrated that the proteins involved in energy metabolism were mostly interacted (Figure 5A).

Twenty DEPs that showed recovery in the mangiferin treated group were selected for further analysis. GO analysis illustrated that most of these proteins were closely related to the metabolic process. KEGG analysis discovered that these proteins were mainly involved in fatty acid degradation, glycolysis, and citrate cycle pathways. STRING analysis showed that HK2, PDHB, ALDH2, LDHA, MDH2, and HADHB closely interacted with other proteins and corresponded to critical nodes in the network (Figure 5B).

### 2.4. Validation of Selected Proteins by Western Blot

**Figure 6:**
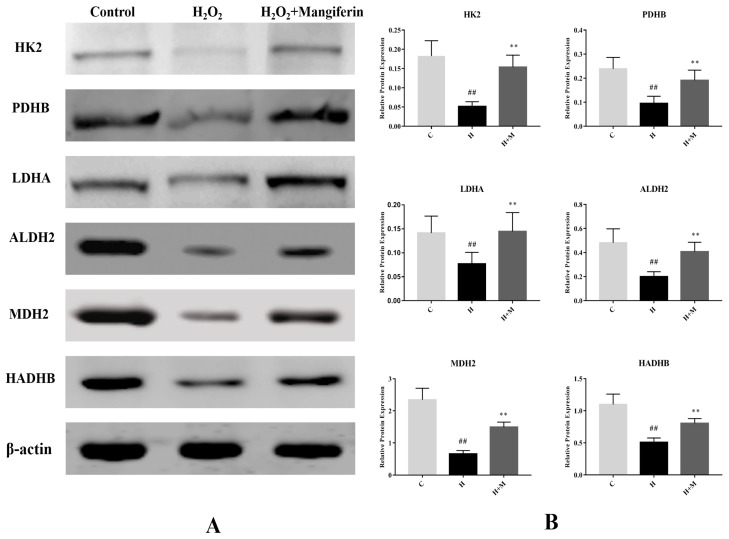
Western blot validation of HK2, PDHB, LDHA, ALDH2, MDH2, and HADHB. ^##^ *p* < 0.01 versus the control; * *p* < 0.05 and ** *p* < 0.01 versus the H_2_O_2_ group.

To further verify the DEPs from the proteomics analysis, the western blot assay was adopted to determine the levels of hexokinase-2 (HK2), pyruvate dehydrogenase E1 component subunit beta (PDHB), aldehyde dehydrogenase (ALDH2), l-lactate dehydrogenase A chain (LDHA), malate dehydrogenase (MDH2), and trifunctional enzyme subunit beta (HADHB) between the three groups. The results elicited that the expression profiles of all the six proteins were downregulated in the H_2_O_2_ group, as compared with the control group, meanwhile all of these proteins were reversed by mangiferin treatment relative to the H_2_O_2_ group (Figure 6). The results of western blot analysis were consistent with the proteomics analysis.

## 3. Discussion

Oxidative stress contributes to the pathogenesis of various cardiovascular diseases, including MIRI [21]. The accumulation of ROS is one of the main causes of myocardial apoptosis [22,23]. The H9C2 cell line is commonly used as a typical cell model to explore the mechanism of cardiovascular disease or the cardiotoxic effects of antitumor drugs [24]. H_2_O_2_ is usually employed to induce H9C2 cell injury in the research of MIRI and oxidative stress injury [7,21,25]. In this research, a cell model of MIRI was successfully reproduced, meanwhile mangiferin attenuated H9C2 cell injury induced by H_2_O_2_ and protected the H9C2 cell line from MIRI.

Under normal physiological conditions, the antioxidant enzymes, including SOD, GSH-Px, and CAT, protect the cells from oxidative injury by eliminating cytoplasmic ROS. However, MIRI will destroy the activities of the antioxidative enzymes [26,27]. SOD catalyzes the disproportionation of the superoxide radical into hydrogen peroxide (H_2_O_2_) [28]. CAT, which exists in the peroxisome of all living organisms, possesses the ability to catalyze the formation of water and oxygen from H_2_O_2_ [29]. GSH-Px catalyzes the decomposition of free hydrogen peroxide to water. MDA is a secondary product of lipid peroxidation and acts as a critical marker for oxidative stress [30]. Our research showed that mangiferin attenuated H9C2 cell injury induced by H_2_O_2_ in a dose-dependent way by enhancing the antioxidative abilities of SOD, CAT, and GSH-Px, and decreasing the content of MDA. These results suggested that mangiferin improved the anti-oxidative activity of the H9C2 cell line.

To elucidate the mechanism underlying mangiferin on the protection of H9C2 injury induced by H_2_O_2_, iTRAQ-based proteomics was conducted to identify the DEPs in the cells. Results of the proteomics analysis showed that most of the DEPs of H9C2 in the H_2_O_2_ group were mostly associated with the metabolic process. Myocardial metabolic disturbances were confirmed to be associated with the pathogenesis of MIRI and other cardiovascular diseases [31,32,33]. Glucose and fatty acids, which act as important substrates for oxidative metabolism, play key roles in the regulation of energy homeostasis in the heart. Oxygen deficit and oxidative stress inhibit the aerobic oxidation of fatty acids and enhance the anaerobic oxidation of glucose [34,35]. Bioinformatics analysis revealed that the DEPs of H9C2 cells in the mangiferin group, which exhibited the tendency to recover, also participated in the metabolic process. Similarly, the KEGG analysis indicated that these recovery DEPs were mostly enriched in glycolysis/gluconeogenesis, pyruvate metabolism, citrate cycle (TCA cycle), and fatty acid degradation. Thus, we hypothesize that mangiferin might ameliorate the H_2_O_2_-induced H9C2 cell injury by regulating glucose and fatty acid metabolism.

Insufficient oxygen shifts the energy metabolism from oxidative phosphorylation to glycolysis in the heart during ischemic conditions. Glycolysis increases after short-time hypoxia, and that is inhibited after long-time hypoxia. Glycolysis is the starting method for aerobic oxidation and anaerobic oxidation of glucose [36,37]. In this study, proteomics research showed that ALDH2, HK2, PDHB, and LDHA, which were related to the glycolysis pathway, significantly changed in the H_2_O_2_ group relative to the control group, whilst all of these four proteins markedly returned to normal in the mangiferin treated group. HK2, a rate-limiting enzyme in glycolysis, catalyzes the formation of glucose-6-phosphate from glucose and regulates the mitochondrial dysfunction in cardiac disease [37,38]. The cardiac HK2, which binds to mitochondria, has been confirmed to protect the cardiomyocytes from death via preventing the mitochondrial permeability transition pore from opening [39]. Previous studies have shown that enhancement of HK2 activity can reduce MIRI injury and H_2_O_2_-induced cell death [40]. PDHB, a main regulator in glycolysis and the citrate cycle, converts pyruvate to acetyl-CoA, which triggers the TCA cycle [41]. The pyruvate dehydrogenase complex plays a pivotal role in the generation of ROS [42]. The activity of pyruvate dehydrogenase complex declines during ischemia [43]. LDHA belongs to the lactate dehydrogenase family, which is known to catalyze the formation of lactate from pyruvate in glycolysis. Prolonged hypoxia results in a reduction of LDHA activity in the heart [44,45]. ALDH2, a member of the aldehyde dehydrogenase family, has been proved to attenuate the toxic aldehydes, maintains mitochondrial function, suppresses ROS production, and influences mitochondrial respiration [46,47]. It has been demonstrated that ALDH2 knockout accelerates the myocardial injury, whilst over-expression of ALDH2 diminishes ROS production and mitigates apoptosis in the cardiomyocytes [48,49]. In this study, the levels of HK2, PDHB, LDHA, and ALDH2 obviously increased in the H_2_O_2_-induced H9C2 cells pretreated with mangiferin relative to the H_2_O_2_ group. These findings indicated that mangiferin attenuated the H9C2 cell injury and oxidative stress induced by H_2_O_2_ through stimulating glycolysis and glucose aerobic oxidation, via increasing the HK2, PDHB, LDHA, and ALDH2 activities.

The TCA cycle is an important hub and links glucose, lipid, and amino acid metabolism. The oxidative stress induced by H_2_O_2_ inhibits the activity of the TCA cycle [50,51]. MDH2 plays a core role in TCA cycle and catalyzes the conversion of malate to oxaloacetate. Previous studies, reported in the literature, reveal that ischemia reduces the activity of MDH2 in the heart [52,53]. The results of proteomics demonstrated that mangiferin could upregulate the reduced activity of MDH2 in the H9C2 cells induced by H_2_O_2_, and, consequently, increased the activity of the TCA cycle.

Insufficient oxygen during ischemia inhibits cardiac fatty acid oxidation and impairs ATP generation regulated by oxidative phosphorylation. Increased fatty acid oxidation promotes the accumulation of cellular free fatty acids in the heart [36]. HADHB catalyzes the last step of fatty acid β-oxidation, which yields acetyl-CoA. The results of this research showed that the levels of HADHB in the cells treated with H_2_O_2_ were significantly lower than those in the control group. Mangiferin markedly increased the activity of HADHB relative to the H_2_O_2_ group. Hence, mangiferin might facilitate the reduced activity of fatty acid β-oxidation by upregulating the level of HADHB.

## 4. Materials and Methods

### 4.1. Reagents and Chemicals

Rat H9C2 cardiomyocyte cell line was purchased from the Cell Bank of the Chinese Academy of Science (Shanghai, China). The mangiferin, ammonium formate, H_2_O_2_, Dimethyl sulfoxide (DMSO), and MTT were all obtained from Sigma-Aldrich (St Louis, MO, USA). Dulbecco’s modified Eagle medium (DMEM) was purchased from Corning Cellgro Inc. (Herndon, VA, USA) and the fetal bovine serum (FBS) was bought from Biological Industries Technologies (Kibbutz Beit Haemek, Israel). The SOD, MDA, GSH-Px, and CAT ELISA assay kits were all acquired from Nanjing Jiancheng Bioengineering Institute (Nanjing, China). The Protein Extraction kit was purchased from Bestbio company (Shanghai, China), and the BCA Protein Assay kit was obtained from CWbiotech Co., Ltd. (Shanghai, China). Trypsin was bought from Promega Corporation (Madison, Wi, USA) and the iTRAQ^®^ Reagents Multiplex kit was obtained from AB Sciex (Redwood, CA, USA). The rabbit primary antibodies of HK2, PDHB, LDHA, ALDH2, MDH2, and HADHB and the secondary antibody were purchased from Bioss company (Beijing, China).

### 4.2. Cell Culture and Oxidative Injury Induced by H_2_O_2_

Cardiomyoblasts H9C2 was cultured in flasks with high glucose Dulbecco’s modified Eagle’s medium (DMEM) supplemented with 10% fetal bovine serum (FBS) containing a mixture of 100 U/mL penicillin and 100 mg/mL streptomycin. The cells were incubated in an atmosphere of 95% air and 5% CO_2_ at 37 °C under humidified conditions. The medium was changed every two or three days. The H9C2 cells were collected with trypsin and seeded in 96-well plates at a density of 1 × 10^5^ cells per well. The cells were used for experiments when they grew to 80–90% confluence. The cells were treated with different concentrations of H_2_O_2_ (0, 50, 100, 200, 300 μM) for 6 h.

### 4.3. Cell Viability Assays

The cell toxicity and viability assays were determined by MTT assay. In brief, the H9C2 cells were seeded in a 96-well plate at a density of 1 × 10^5^ cells per well and cultured for 24 h. Mangiferin was dissolved in DMEM with 0.1% DMSO. The DMEM was removed and different concentrations of mangiferin (0, 5, 10, 20, 50, 100, 200, 400, 800 μM) were added in the wells and incubated for 6 h. To determine the protective effect of mangiferin on H_2_O_2_-induced cell injury, the H9C2 cardiomyocytes were preconditioned with 5, 10, and 20 μM/L mangiferin for 6 h, followed by treatment with H_2_O_2_ for 6 h. Subsequently, the cells were incubated with 10 μL MTT (0.5 mg/mL) in each well. After incubation at 37 °C for 4 h, the supernatants were discarded, and 100 μL DMSO was added in each well to dissolve the formazan crystal, after which the absorbance was determined with a microplate reader at 490 nm.

### 4.4. Biochemical Analysis of H9C2 Cells

To determine the antioxidant effect of mangiferin, the SOD, MDA, CAT, and GSH-Px levels were measured by the ELISA kits in accordance with the manufacture’s instruments.

### 4.5. Protein Preparation and iTRAQ Labelling

H9C2 cells were seeded in the 150 mm Petri dishes with a density of 2 × 10^4^/cm^2^, which were incubated up to 80–90% confluence for the iTRAQ-based proteomics analysis. According to the results of the cell viability and biochemical assays, a high dose (20 μM/L) of mangiferin was selected for the proteomics research. H9C2 cells were divided into six groups including two control groups, two H_2_O_2_ groups, and two H_2_O_2_ + mangiferin groups. The cells in the control group were cultured in normal condition, the cells in the H_2_O_2_ group were incubated with H_2_O_2_ (200 μM) for 6 h, and the cells in the H_2_O_2_ + mangiferin groups were pretreated with mangiferin (20 μM/L) for 6 h, and subsequently cultured with H_2_O_2_ (200 μM) for 6 h. After incubation in H_2_O_2_, the medium was discarded from each cultured dish and rinsed with cold PBS twice. The cell proteins were extracted by the protein extraction assay kit and the protein concentrations were measured by the BCA protein assay kit, all the procedures were followed as to the manufacturers’ instructions.

The extracted proteins (100 μg) from each group were digested in trypsin at 37 °C overnight, then the peptides were labeled with iTRAQ reagent-8-plex kit in accordance with the manufacturer’s instruction. Peptides in the control group were labeled with 115 and 118, meanwhile the peptides of the H_2_O_2_ group were labeled with 116 and 119, and the peptides in H_2_O_2_ + mangiferin group were labeled with 117 and 121. All the labeled peptides were mixed together and vacuum dried.

### 4.6. High-pH Reverse-Phase Liquid Chromatography Fraction

The mixture of labeled peptides were separated on an HPLC system (Waters, Milford, MA, USA) combined with the Waters XBridge C18 (4.6 × 250 mm, 5 μm). The dried peptides were dissolved in buffer A (20 mM ammonium formate, pH 10), and vortexed at 12,000× *g* for 20 min. The supernatant was loaded onto the HPLC column. The peptides were eluted at a 1 mL/min with a gradient of 5% buffer B (20 mM ammonium formate in 80% acetonitrile, pH 10) for 5 min, 5–15% buffer B for 25 min, 15–38% buffer B for 15 min, 38–90% buffer B for 1 min, and 90% buffer B continuously eluted for a period of 8.5 min, 90–5% buffer B for 0.5 min, and 5% buffer B for 10 min. The eluted fractions were collected after 5 min at 1 min intervals and divided into ten fractions. All ten fractions were dried by vacuum centrifugation.

### 4.7. Nano LC-MS/MS Analysis

Nano LC-MS/MS analysis was carried out on an AB SCIEX nanoLC-MS/MS (Triple TOF 5600) system (Redwood, CA, USA). All the ten fractions were dissolved in 30 μL of mobile phase A (0.1% formic acid, 2% acetonitrile) and centrifuged at 12,000× *g* for 20 min. Afterwards, 20 μL of each samples was separated in an analytical column (C18-CL-120, 0.075 × 150 mm, 3 μm) with an 80 min solvent gradient from 5–80% buffer B (0.1% formic acid, 98% acetonitrile) at a flow rate of 0.3 μL/min. MS1 spectra were collected in the range 350–1250 *m/z* for 250 ms. The 30 precursors with the strongest signals were selected for fragmentation. MS2 spectra were collected in the range of 100–1500 *m/z* for 100 ms.

### 4.8. Protein Identification and Bioinformatic Analysis

The AB ProteinPilot™ v4.5 software (AB SCIEX, Redwood, CA, USA) was applied to analyze the MS data for protein identification with the Paragon algorithm. The proteins that possessed at least one unique peptide and more than 1.3 unused values were chosen for further identification, and proteins which possessed at least two peptides were considered for further analysis. *p* ≤ 0.05 and fold-changes lower than 0.67 or higher than 1.5 were considered as significant.

Functional classification and annotation were performed with GO using the DAVID bioinformatics resources 6.8 (http://david.ncifcrf.gov/) to determine enrichment in CC, BP, and MF. Pathway analysis was performed using the KEGG (http://www.genome.ad.jp/kegg/mapper.html) to identify significant enrichment of signal pathways. STRING version 11.0 (https://string-db.org/) was employed to explore the protein interaction networks.

### 4.9. Western Blot Validation

The levels of HK2, PDHB, LDHA, ALDH2, MDH2, and HADHB were validated by western blot analysis. In brief, an equal amount of protein (30 μg) from each group was separated with 12% SDS-PAGE gels and transferred to 0.22 μM NC membranes, followed by being blocked with the QuickBlock blocking buffer for 15 min. The blocked membranes were washed with PBST three times, and all the primary antibodies at a dilution of 1:1000 were added to the membrane and incubated at 4 °C overnight. Afterwards, the membranes were washed with PBST and incubated with the secondary anti-rabbit antibody at a dilution of 1:5000 for 1 h. After being washed with PBST, the bands were detected by the Odyssey Imaging System (LI-COR Bioscience, Inc., Lincoln, USA).

### 4.10. Statistical Analysis

Data were expressed as mean ± SD. Statistical analysis was performed on GraphPad Prism software (GraphPad Software, Inc., La Jolla, CA, USA). One-way ANOVA and Student’s *t*-test were used as appropriate. *p* values of less than 0.05 were considered statistically significant.

## 5. Conclusions

In this study, iTRAQ-based proteomics was employed to elucidate the underlying mechanism of mangiferin against MIRI induced by H_2_O_2_ in vitro for the first time. In conclusion, mangiferin could exert its protective effect on oxidative-stress-induced MIRI by enhancing the antioxidative capacity of the H9C2 cell, as well as promoting glucose aerobic metabolism and fatty acid oxidation via activating the glycolysis, citrate cycle, and fatty acid degradation pathways. Subsequent validation proved that HK2, PDHB, LDHA, ALDH2, MDH2, and HADHB could be selected as the candidate targets of mangiferin in myocardial protection. Our findings provided novel insights for the prevention of MIRI and elucidated the mechanism of mangiferin in myocardial protection.

## Supplementary Materials

The following are available online. Table S1: The differently-expressed proteins in the H_2_O_2_ group versus the control group, Table S2: The differently-expressed proteins in the mangiferin group versus the H_2_O_2_ group.

## Abbreviations

ALDH2	Aldehyde dehydrogenase
CAT	Catalase
DMSO	Dimethyl sulfoxide
GSH-Px	Glutathione peroxidase
H_2_O_2_	Hydrogen peroxide
HADHB	Trifunctional enzyme subunit beta
HK2	Hexokinase-2
LDHA	L-lactate dehydrogenase A chain
MDA	Malonaldehyde
MDH2	Malate dehydrogenase
MTT	3-(4,5-Dimethylthiazol-2-yl)-2,5-diphenyltetrazolium bromide
PDHB	Pyruvate dehydrogenase E1 component subunit beta
SOD	Superoxide dismutase

## References

[1] Song L.N., Yang H., Wang H.X., Tian C., Liu Y., Zeng X.J., Gao E., Kang Y.M., Du J., Li H.H. (2014). Inhibition of 12/15 lipoxygenase by baicalein reduces myocardial ischemia/reperfusion injury via modulation of multiple signaling pathways. Apoptosis.

[2] Hausenloy D.J., Yellon D.M. (2013). Myocardial ischemia-reperfusion injury: A neglected therapeutic target. J. Clin. Invest..

[3] Kalogeris T., Bao Y.M., Korthuis R.J. (2014). Mitochondrial reactive oxygen species: A double edged sword in ischemia/reperfusion vs preconditioning. Redox Biol..

[4] Turer A., Hill J. (2010). Pathogenesis of myocardial ischemia-reperfusion injury and rationale for therapy. J. Am. Coll. Cardiol..

[5] Loke K.E., McConnell P.I., Tuzman J.M., Shesely E.G., Smith C.J., Stackpole C.J., Thompson C.I., Kaley G., Wolin M.S., Hintze T.H. (1999). Endogenous endothelial nitric oxide synthase–derived nitric oxide is a physiological regulator of myocardial oxygen consumption. Circ. Res..

[6] Xia Y., Zweier J.L. (1995). Substrate control of free radical generation from xanthine oxidase in the postischemic heart. J. Biol. Chem..

[7] Chou H.C., Chen Y.W., Lee T.R., Wu F.S., Chan H.T., Lyu P.C., Timms J.F., Chan H.L. (2010). Proteomics study of oxidative stress and Src kinase inhibition in H9C2 cardiomyocytes: A cell model of heart ischemia–reperfusion injury and treatment. Free Radic. Biol. Med..

[8] Zhao X., Dou M., Zhang Z., Zhang D., Huang C. (2017). Protective effect of Dendrobium officinale polysaccharides on H2O2-induced injury in H9c2 cardiomyocytes. Biomed. Pharmacother..

[9] Liu Y.W., Cheng Y.Q., Liu X.L., Hao Y.C., Li Y., Zhu X., Zhang F., Yin X.X. (2017). Mangiferin upregulates glyoxalase 1 through activation of Nrf2/are signaling in central neurons cultured with high glucose. Mol. Neurobiol..

[10] Muruganandan S., Gupta S., Kataria M., Lal J., Gupta P. (2002). Mangiferin protects the streptozotocin-induced oxidative damage to cardiac and renal tissues in rats. Toxicology.

[11] Miura T., Ichiki H., Iwamoto N., Kato M., Kubo M., Sasaki H., Okadia M., Ishida T., Seino Y., Tanigawa K. (2001). Antidiabetic activity of the rhizoma of Anemarrhena asphodeloides and active components, mangiferin and its glucoside. Biol. Pharm. Bull..

[12] Miura T., Ichiki H., Hashimoto I., Iwamoto N., Kao M., Kubo M., Ishihara E., Komatsu Y., Okada M., Ishida T. (2001). Antidiabetic activity of a xanthone compound, mangiferin. Phytomedicine.

[13] Telang M., Dhulap S., Mandhare A., Hirwani R. (2013). Therapeutic and cosmetic applications of mangiferin: A patent review. Expert Opin Ther Pat..

[14] Pal P.B., Sinha K., Sil P.C. (2014). Mangiferin Attenuates Diabetic Nephropathy by Inhibiting Oxidative Stress Mediated Signaling Cascade, TNFα Related and Mitochondrial Dependent Apoptotic Pathways in Streptozotocin-Induced Diabetic Rats. PLoS ONE.

[15] Shi C.X., Li X.J., Yuan L.L., Cheng B.H., Xu X.D. (2017). Protective effects of mangiferin on the models of BRL cell oxidative stress induced by H2O2. Anat. Res..

[16] Chao W., Xian W.G. (2008). Cardioprotective Effects of Mangiferin on Myocardial in Schemia Reperfusion Injury in Rats. Chin. J. Arterioscler..

[17] Suchal K., Malik S., Gamad N., Malhotra R.K., Goyal S.N., Ojha S., Kumari S., Bhatia J., Arya D.S. (2016). Mangiferin protect myocardial insults through modulation of MAPK/TGF-β pathways. Eur. J. Pharmacol..

[18] Mirza S.P. (2012). Quantitative mass spectrometry-based approaches in cardiovascular research. Circ.-Cardiovasc. Gene..

[19] Mesaros C., Blair I.A. (2016). Mass spectrometry-based approaches to targeted quantitative proteomics in cardiovascular disease. Clin. Proteomics..

[20] Mokou M., Lygirou V., Vlahou A., Mischak H. (2017). Proteomics in cardiovascular disease: Recent progress and clinical implication and implementation. Expert Rev. Proteomic..

[21] Sun G., Ye N., Dai D., Chen Y., Li C., Sun Y. (2016). The protective role of the TOPK/PBK pathway in myocardial ischemia/reperfusion and H2O2-induced injury in H9C2 cardiomyocytes. Int. J. Mol. Sci..

[22] Kumar D., Jugdutt B.I. (2003). Apoptosis and oxidants in the heart. J. Lab. Clin. Med..

[23] Circu M.L., Aw T.Y. (2010). Reactive oxygen species, cellular redox systems, and apoptosis. Free Radic. Biol. Med..

[24] Lenčo J., Lenčová-Popelová O., Link M., Jirkovská A., Tambor V., Potůčková E., Stulík J., Šimůnek T., Štěrba M. (2015). Proteomic investigation of embryonic rat heart-derived H9c2 cell line sheds new light on the molecular phenotype of the popular cell model. Exp. Cell Res..

[25] Xu J., Tang Y., Bei Y., Ding S., Che L., Yao J., Wang H., Lv D., Xiao J. (2016). miR-19b attenuates H2O2-induced apoptosis in rat H9C2 cardiomyocytes via targeting PTEN. Oncotarget.

[26] Mates J. (2000). Effects of antioxidant enzymes in the molecular control of reactive oxygen species toxicology. Toxicology.

[27] Vijayasarathy K., Naidu K.S., Sastry B. (2010). Melatonin metabolite 6-Sulfatoxymelatonin, Cu/Zn superoxide dismutase, oxidized LDL and malondialdehyde in unstable angina. Int. J. Cardiol..

[28] Sheng Y., Abreu I.A., Cabelli D.E., Maroney M.J., Miller A.-F., Teixeira M., Valentine J.S. (2014). Superoxide dismutases and superoxide reductases. Chem Rev..

[29] Chelikani P., Fita I., Loewen P.C. (2004). Diversity of structures and properties among catalases. Cell Mol. Life Sci..

[30] Nielsen F., Mikkelsen B.B., Nielsen J.B., Andersen H.R., Grandjean P. (1997). Plasma malondialdehyde as biomarker for oxidative stress: Reference interval and effects of life-style factors. Clin. Chem..

[31] Lopaschuk G. (2000). Regulation of carbohydrate metabolism in ischemia and reperfusion. Am. Heart J..

[32] Kyu Kim H., Thu V.T., Heo H.J., Kim N., Han J. (2011). Cardiac proteomic responses to ischemia–reperfusion injury and ischemic preconditioning. Expert Rev. Proteomic..

[33] Xu Z.W., Chen X., Jin X.H., Meng X.Y., Zhou X., Fan F.X., Mao S.Y., Wang Y., Zhang W.C., Shan N.N. (2016). SILAC-based proteomic analysis reveals that salidroside antagonizes cobalt chloride-induced hypoxic effects by restoring the tricarboxylic acid cycle in cardiomyocytes. J. Proteomics..

[34] Von Lewinski D., Gasser R., Rainer P.P., Huber M.S., Wilhelm B., Roessl U., Haas T., Wasler A., Grimm M., Bisping E. (2010). Functional effects of glucose transporters in human ventricular myocardium. Eur. J. Heart Fail..

[35] Lopaschuk G.D., Belke D.D., Gamble J., Toshiyuki I., Schönekess B.O. (1994). Regulation of fatty acid oxidation in the mammalian heart in health and disease. Biochim. Biophys. Acta (BBA)-Lipids Lipid Metab..

[36] Oliver E.F., Opie L. (1994). Effects of glucose and fatty acids on myocardial ischaemia and arrhythmias. Lancet.

[37] Zhang T.J., Guo R.X., Li X., Wang Y.W., Li Y.J. (2017). Tetrandrine cardioprotection in ischemia–reperfusion (I/R) injury via JAK3/STAT3/Hexokinase II. Eur. J. Pharmacol..

[38] Wilson J.E. (2003). Isozymes of mammalian hexokinase: Structure, subcellular localization and metabolic function. J. Exp. Biol..

[39] Miyamoto S., Murphy A.N., Brown J.H. (2008). Akt mediates mitochondrial protection in cardiomyocytes through phosphorylation of mitochondrial hexokinase-II. Cell Death Differ..

[40] Zuurbier C.J., Eerbeek O.O., Meijer A.J. (2005). Ischemic preconditioning, insulin and morphine all cause hexokinase redistribution. Am. J. Physiol-Heart C..

[41] Daisuke K., Yoji Andrew M., Koh N. (2014). Prolyl-hydroxylase PHD3 interacts with pyruvate dehydrogenase (PDH)-E1β and regulates the cellular PDH activity. Biochem. Biophys. Res. Commun..

[42] Quinlan C.L., Goncalves R.L.S., Martin H.M., Nagendra Y., Bunik V.I., Brand M.D. (2014). The 2-oxoacid dehydrogenase complexes in mitochondria can produce superoxide/hydrogen peroxide at much higher rates than complex I. J. Biol. Chem..

[43] Kobayashi K., Neely J.R. (1983). Effects of ischemia and reperfusion on pyruvate dehydrogenase activity in isolated rat hearts. J. Mol. Cell Cardiol..

[44] Zhang Y., Liu G., Gao X. (2017). Attenuation of miR-34a protects cardiomyocytes against hypoxic stress through maintenance of glycolysis. Biosci Rep..

[45] Luo C., Wang H., Chen X., Cui Y., Li H., Long J., Mo X., Liu J. (2013). Protection of H9c2 rat cardiomyoblasts against oxidative insults by total paeony glucosides from Radix Paeoniae Rubrae. Phytomedicine.

[46] Luo X.J., Liu B., Ma Q.L., Peng J. (2014). Mitochondrial aldehyde dehydrogenase, a potential drug target for protection of heart and brain from ischemia/reperfusion injury. Curr. Drug Targets..

[47] Mali V.R., Deshpande M., Pan G., Thandavarayan R.A., Palaniyandi S.S. (2016). Impaired ALDH2 activity decreases the mitochondrial respiration in H9C2 cardiomyocytes. Cell Signal..

[48] Wang H., Kang P., Hongwei Y.E., Ying Y.U., Wang X., Qin G. (2012). Anti-apoptotic role of mitochondrial aldehyde dehydrogenase 2 in myocardial ischemia/reperfusion injury in diabetic rats. South Med. J..

[49] Hussain C., Nicolaj B., Colin C., Cedric M., Jonathan D., Ormerod J.O.M., Rebekka J., Hans Erik B.T., Andrew R., Schmidt M.R. (2013). Aldehyde dehydrogenase-2 inhibition blocks remote preconditioning in experimental and human models. Basic Res. Cardiol..

[50] Janero D.R., Hreniuk D. (1996). Suppression of TCA cycle activity in the cardiac muscle cell by hydroperoxide-induced oxidant stress. Am. J. Physiol..

[51] Tretter L., Adam V.V. (2000). Inhibition of Krebs cycle enzymes by hydrogen peroxide: A key role of [alpha]-ketoglutarate dehydrogenase in limiting NADH production under oxidative stress. J. Neurosci..

[52] Lu W.D., Qiu J., Zhao G.X., Qie L.Y., Wei X.B., Gao H.Q. (2012). Quantitative Mitochondrial Proteomics Study on Protective Mechanism of Grape Seed Proanthocyanidin Extracts Against Ischemia/Reperfusion Heart Injury in Rat. Chem. Res. Chin. Univ..

[53] Kim N., Lee Y., Kim H., Joo H., Youm J.B., Park W.S., Warda M., Cuong D.V., Han J. (2010). Potential biomarkers for ischemic heart damage identified in mitochondrial proteins by comparative proteomics. Proteomics.

